# Emetine elicits apoptosis of intractable B-cell lymphoma cells with *MYC* rearrangement through inhibition of glycolytic metabolism

**DOI:** 10.18632/oncotarget.14393

**Published:** 2016-12-31

**Authors:** Tomohiro Aoki, Kazuyuki Shimada, Akihiko Sakamoto, Keiki Sugimoto, Takanobu Morishita, Yuki Kojima, Satoko Shimada, Seiichi Kato, Chisako Iriyama, Shunsuke Kuno, Yasuhiko Harada, Akihiro Tomita, Fumihiko Hayakawa, Hitoshi Kiyoi

**Affiliations:** ^1^ Department of Hematology and Oncology, Nagoya University Graduate School of Medicine, Nagoya, Japan; ^2^ Institute for Advanced Research, Nagoya University, Nagoya, Japan; ^3^ Fujii Memorial Research Institute, Otsuka Pharmaceutical Co., Ltd., Otsu, Japan; ^4^ Department of Pathology and Clinical Laboratories, Nagoya University Hospital, Nagoya, Japan; ^5^ Department of Mechanism of Aging, National Center for Geriatrics and Gerontology, Obu, Japan; ^6^ Department of Hematology, Japanese Red Cross Nagoya Daiichi Hospital, Nagoya, Japan; ^7^ Department of Hematology, Fujita Health University School of Medicine, Toyoake, Japan

**Keywords:** diffuse large B-cell lymphoma, emetine, glycolysis, high-throughput drug screening, microenvironment

## Abstract

Despite improved clinical outcomes of diffuse large B-cell lymphoma, a certain proportion of patients still develop a primary refractory disease. To overcome these lymphomas that are intractable to existing treatment strategies, the tumor microenvironment has been identified as a potential therapeutic target. Here we describe our search for effective drugs for primary refractory lymphoma cells with *MYC* rearrangement. Through the drug screening of 3,440 known compounds, we identified a unique compound, emetine. This compound was effective against lymphoma cells with *MYC* rearrangement from two different patients that were co-cultured with cancer associated fibroblasts. Emetine induced the death of these cells with a half maximal inhibitory concentration of 312 nM and 506 nM, respectively. Subsequent analyses of the mechanism of action of emetine showed that the drug induced apoptosis of tumor cells via alteration of glucose metabolism through inhibition of hypoxia inducible factor-1α. Moreover, emetine inhibited the potential of cancer associated fibroblasts to support tumor cell viability *in vitro* and demonstrated significant inhibition of tumor growth in *in vivo* analyses. Emetine also induced cell death in other primary refractory lymphoma cells with *MYC* rearrangement. Our combined data indicate that emetine is a potential promising drug for the treatment of intractable lymphomas, which targets both the tumor and its microenvironment.

## INTRODUCTION

Diffuse large B-cell lymphoma (DLBCL) is a heterogeneous disorder comprising 25-30% of non-Hodgkin lymphomas [1]. The R-CHOP (rituximab, cyclophosphamide, doxorubicin, vincristine and prednisolone) regimen has markedly improved outcomes in DLBCL over the last decade. However, the prognosis of DLBCL with *MYC* rearrangement, which regulates multiple functions including cell cycle progression, cell proliferation, apoptosis, and glucose metabolism, remains poor with a median overall survival of less than 1 year [2–10]. Although intensive induction regimens and/or targeting treatment approaches that directly or indirectly interfere with MYC function including targeting of mTOR, PI3K or NF-κB have been developed, [11–15] these approaches failed to show a benefit in the relevant clinical trials [3, 16, 17]. Therefore, innovative approaches for the development of novel therapies are vital in order to improve outcomes in DLBCL patients with *MYC* rearrangement.

Recent findings suggest that resistance to chemotherapy is mediated by interactions between the tumor cells and their microenvironment [18–20]. The tumor microenvironment has therefore drawn much attention as an attractive potential therapeutic target for intractable lymphoma [20, 21]. For example, it has been shown that stromal cells in the tumor microenvironment can promote a metabolic switch in malignant tumor cells away from mitochondrial respiration to glycolysis [22]. This so-called Warburg effect confers growth advantages and drug resistance to tumors [23].

Here, we report regarding the discovery of a novel therapy targeting the tumor microenvironment to overcome the poor prognosis of intractable DLBCL with *MYC* rearrangement. We applied primary patient lymphoma cells that were co-cultured with cancer associated fibroblasts (CAF) derived from a human lymph node to a previously reported high throughput drug screening system [24] and identified an effective anti-tumor drug, emetine. We also elucidated a novel mechanism of emetine *in vitro* and *in vivo*, i.e., emetine induced the apoptosis of tumor cells via inhibition of glycolysis. Our results indicate that emetine, which was discovered with a screening method, may be a promising drug for targeting the tumor of intractable lymphoma and its microenvironment.

## RESULTS

### Establishment of the *in vitro* culture system for primary lymphoma cells

**Figure 1:**
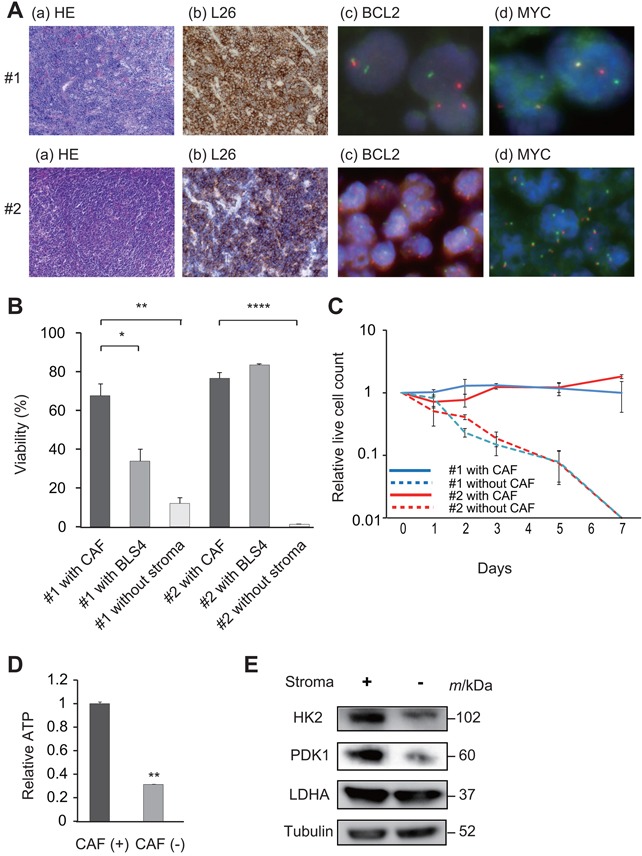
Establishment of *ex vivo* culture of primary lymphoma cells using CAF **A**. Pathological specimens of lymph node samples of intractable DLBCL patients (#1 and #2). HE staining (a), L26 immunostaining (b), split FISH assays for *BCL2* probes (c) and for *MYC* probes (d) are shown. **B**. Viability of lymphoma cells at 48 h after initiation of co-culture with or without CAF. A bar graph of relative cell viability under each culture condition is shown. Each point represents the mean value taken from three representative independent experiments. Error bars indicate SEM. Asterisks indicate the *P* value as follows; * *P* < 0.05; ** *P* < 0.01; **** *P* < 0.0001. **C**. Long-term *ex vivo* culture of lymphoma cells. Lymphoma cells of patients #1 and #2 were cultured with or without CAF. Each point represents the mean value taken from three representative independent experiments. Error bars indicate SEM. **D**. ATP levels were measured in lysates from lymphoma cells (#2) cultured with or without CAF. Each point represents the mean value taken from three representative independent experiments. Error bars indicate SEM. Asterisks indicate the *P* value as follows; ** *P* < 0.01 **E**. Whole cell lysates of tumor cells (#2) were obtained at 48 h after initiation of the co-culture with or without CAF. Immunoblotting was performed for HK2, PDK1 and LDHA. Tubulin was blotted as a loading control.

We encountered primary refractory DLBCL patients with *MYC* rearrangement during our usual clinical practice. The detailed clinical characteristics of the two patients who demonstrated resistance to conventional immunochemotherapies and whose tumor cells we analyzed are shown in Table 1. Both patients developed refractory diseases within 1 year after diagnosis that were accompanied by *MYC* and *BCL2* rearrangements in their tumor cells. These rearrangements were detected via break-apart fluorescence in-situ hybridization (FISH) that was performed using their formalin-fixed paraffin-embedded (FFPE) tumor tissue (Figure 1A). To search for drugs effective against these intractable DLBCL tumors, we performed high-throughput drug screening using a library that mainly contained known pharmacologically active substances or off-patent drugs.

**Table 1:** Characteristics of DLBCL patients #1 and #2

Name	#1	#2
Origin of cells	Lymph node	Peripheral blood
Age	29	75
*MYC* rearrangement	+	+
*BCL2* rearrangement	-	+
MYC protein in IHC	-	+
BCL2 protein in IHC	+	+
CNS invasion	+	+
1st line treatment	DA-EPOCH-R	R-CHOP
Response	Progressive disease	Partial response
Overall Survival (Mo)	5	31
Progression-free Survival (Mo)	1	10
Cytogenetic analyses	46, XY, add(1)(q21), add (3)(p13), add(4)(p16),?t(8;22)(q24;q11.2), add(17)(p11.2)	48, XX, +X, add(1)(p36.1), add (5)(q31),add(7)(q22), t(8;14)(q24;q32), del(13)(q?), t(14;18)(q32.q21),+der(18)t(14;18),-22, -22,+der(?)t(?.q21),+mar1

We recently reported that the mouse fibroblastic reticular cell line BLS4, which was established from a mouse lymph node, [25] provides glutathione to tumor cells, and enables the culture of patient-derived xenograft (PDX) lymphoma cells *in vitro* [24]. However, to perform drug screening against primary patient lymphoma cells, we considered that it was important to use an *in vitro* co-culture system based on human origin cells. We therefore cultured lymph node samples from lymphoma patients, and successfully isolated stromal cells that are described in detail in the Materials and Methods section. The surface phenotype of these stromal cells, i.e., α-smooth muscle actin (SMA) positive and CD31 negative, were coincident with those of fibroblasts, and these cells were therefore considered to be CAF. We then investigated whether we could *in vitro* culture patient lymphoma cells on the fastest-growing of these CAF. In the subsequent screening analyses, primary tumor cells obtained from the lymph node biopsy of patient (Pt) #1 were used. Tumor cells from a PDX mouse model were used for Pt #2 to validate the results for Pt #1, as we were unable to obtain a large number of primary tumor cells from the lymph node biopsy of Pt #2. Although lymphoma cells from neither of the patients could survive for a long period in an *in vitro* monoculture, they could survive in co-culture with CAF for a much longer time than in monoculture (Figure 1B and 1C). Intriguingly, the viability of the tumor cells of Pt #1 that were co-cultured with CAF was significantly superior to that of the cells co-cultured with BLS4, whereas the viability of the tumor cells of Pt #2 from the PDX model did not differ between co-culture with stromal cells of CAF or BLS4 (Figure 1B and 1C). Primary lymphoma cells without *MYC* rearrangement could also survive in co-culture with CAF for a longer time than in monoculture (Supplemental Figure S1A). We next analyzed adenosine triphosphate (ATP) production in the tumor cells based on a previous finding that stromal cells induced an increase in ATP in tumor cells, resulting in cell proliferation and survival [22]. As expected, ATP increased in tumor cells that were co-cultured with CAF (Figure 1D). Moreover, since the tumor environment promotes glycolysis resulting in energy production in tumor cells, [22] we analyzed the expression of key enzymes involved in glycolysis including hexokinase-2 (HK2), pyruvate dehydrogenase kinase-1 (PDK1), and lactate dehydrogenase A (LDHA) in the presence or absence of co-culture with CAF, using western blotting. Markedly increased expression of HK2 and PDK1 was observed when the cells were co-cultured with CAF (Figure 1E). These combined data indicated that CAF isolated from human lymph nodes promotes the glycolysis of tumor cells, which allows evaluation of the survival of primary tumor cells in an *in vitro* co-culture system.

### High throughput drug screening using intractable lymphoma cells co-cultured with CAF

**Figure 2:**
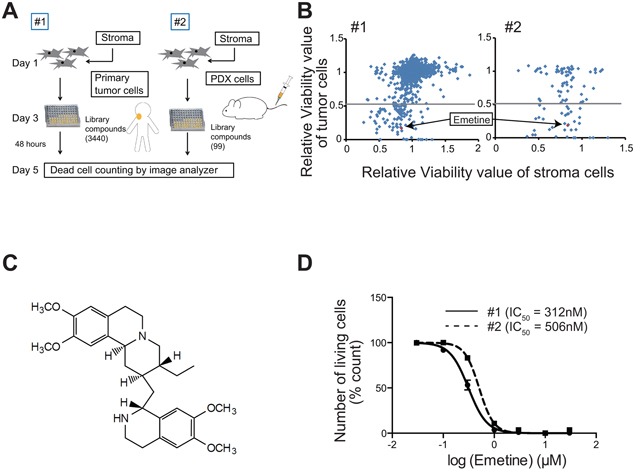
High-throughput drug screening for intractable lymphoma **A**. The scheme of the drug screening method is shown. Library compounds were added to lymphoma cells that were co-cultured with CAF on 96-well plates. After 48 h, the viability of the lymphoma cells was analyzed using an image analyzer. **B**. Results of the drug screening are shown. All compounds were plotted on a scattergram in which the relative viability of lymphoma cells and of CAF were indicated on the Y-axis and X-axis, respectively. Emetine is indicated as a red square. The gray line indicates a relative viability value of 0.5. **C**. The chemical structural formula of emetine is shown. **D**. Dose-dependent anti-lymphoma effects of emetine. The death of lymphoma cells from patient #1 (circles, solid line) and #2 (squares, dotted-line) that were co-cultured with CAF in the presence of various concentrations of emetine for 48 h is shown. Each point represents the mean value for three independent experiments. Error bars represent SEM.

To search for a drug compound that is effective against the tumor cells, we performed drug screening using the primary tumor cells from Pt #1 that were co-cultured with CAF (Figure 2A). The results for all 3,440 compounds were plotted on scattergrams, in which the viability of the primary tumor cells was indicated on the Y-axis, and the WST-8 values of the CAF were indicated on the X-axis (Figure 2B). Ninety-nine compounds for which the viability of tumor cells was less than 0.5 were identified as potentially effective compounds. Subsequently, we validated the effect of these 99 compounds by testing them against the lymphoma cells from Pt #2 that were derived from the PDX model. Ultimately 10 compounds were identified as potentially effective for both of the primary lymphomas (Figure 2B and Supplemental Table S1). Among these potentially effective drugs, several drugs including Verteporfin and Brefeldin A are known to display high toxicities and poor bioavailability in humans. In addition, the drug dose of 2 μM that was used in the current screening was too high for the use of anthracyclines in humans, considering the equivalent concentration in humans. Taking these factors, as well as the possibility of translation to the clinic for the remaining drugs into account, emetine was therefore chosen from these 10 compounds for further analyses (Figure 2C). We confirmed that emetine induced the death of lymphoma cells from both Pt #1 and #2; the half maximal inhibitory concentration (IC_50_) value was 312 nM and 506 nM, respectively (Figure 2D). Moreover, emetine also induced the death of lymphoma cells without *MYC* rearrangement with a moderately higher IC_50_ value (Supplemental Figure S1B), and strongly inhibited the growth of various lymphoma cell lines with a GI_50_ ranging from 1.5 to 321 nM (Supplemental Figure S1C).

### The effects of emetine on CAF

**Figure 3:**
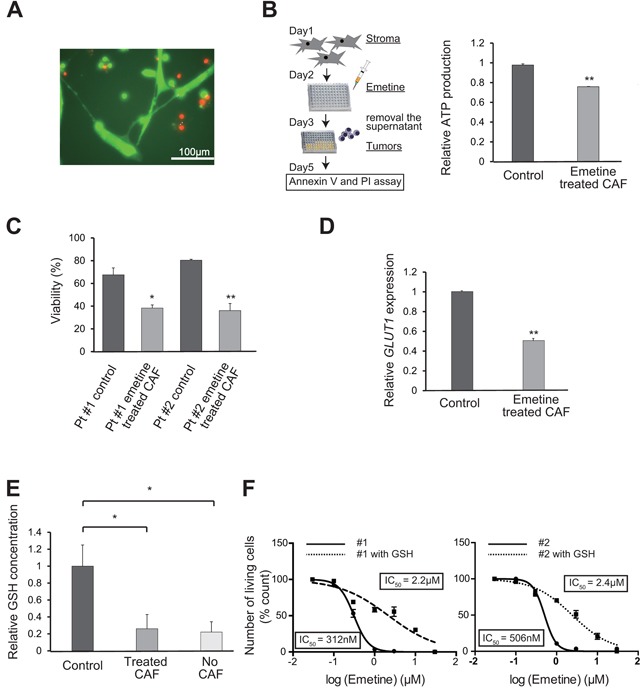
The mechanism of action of emetine on CAF **A**. Fluorescent microscopic analysis of lymphoma cells and CAF treated with emetine. Lymphoma cells (#2) co-cultured with CAF were assessed after treatment with 2 μM emetine for 48 h. Live and dead cells stained with Calcein-AM (green) and PI (red), respectively, are shown. **B**. ATP production in lymphoma cells co-cultured with emetine-pre-treated CAF. The scheme of the experiment is shown at left.ATP levels were assessed in lysates from lymphoma cells (#2) (3 × 10^4^ cells per well) co-cultured with non-treated (Control) CAF or with CAF that were treated with emetine (3 × 10^3^ cells per well, 0.5 μM, for 48 h) prior to initiation of the co-culture. Each point represents the mean value taken from three independent experiments. Error bars indicate SEM. **C**. Assessment of the death of lymphoma cells co-cultured with non-treated or emetine-pre-treated CAF. The scheme of the experiment is shown at left in (B). The death of lymphoma cells (#1 and #2) was assessed 48 h after co-culture with non-treated or emetine-pre-treated CAF. Each point represents the mean value taken from two independent experiments. Error bars indicate SEM. Asterisks indicate the *P* value as follows; * *P* < 0.05; ** *P* < 0.01. **D**. Relative gene expression of the glucose transporter *GLUT1*. RNA was extracted 48 h after co-culture of lymphoma cells (#2) with non-treated or emetine-pre-treated CAF, and relative mRNA expression of *GLUT1* was then assessed using quantitative RT-PCR. Each point represents the mean value taken from two independent experiments. Error bars indicate SEM. Asterisks indicate the *P* value as follows; ** *P* < 0.01. **E**. Intracellular GSH concentration of lymphoma cells (#2) co-cultured with non-treated (control) or emetine-pre-treated CAF, or cultured as a monoculture (No CAF). Each point represents the mean value taken from three independent experiments. Error bars indicate SEM. Asterisks indicate the *P* value as follows; * *P* < 0.05. **F**. Escape from the anti-tumor effect of emetine by the addition of GSH into the culture medium. Cell death of lymphoma cells (#1, left and #2, right) co-cultured with CAF in the presence of various concentrations of emetine supplemented with (squares, dotted-line) or without (circles, solid line) 2 mM GSH for 48 h is shown. Each point represents the mean value taken from three independent experiments. Error bars indicate SEM.

To understand the underlying biology of emetine, we first investigated the effect of emetine on CAF. The proliferation and survival of CAF were not decreased when treated with emetine at the concentration of emetine (2 μM) that was used for the screening (Figure 3A and Supplemental Figure S2A and S2B). Next, to evaluate the effect of emetine on the ability of CAF to support tumor growth, we pretreated CAF with or without emetine for 48 h and then washed the CAF to remove the drug prior to initiation of the co-culture. Both ATP production and CAF support of tumor cell survival decreased in tumor cells co-cultured with CAF that were pretreated with 0.5 μM emetine (Figure 3B and 3C). This decreased support of tumor cell survival was observed in the absence of a direct contact between the tumor cells and CAF, when the tumor cells and CAF were separated by a transwell with a pore size of 0.4 μm (Supplemental Figure S2C). This result implied that CAF support of cell survival was mediated by small molecules including metabolites, cytokines and microvesicles. The mRNA expression of *GLUT1*, which is an indicator of glucose metabolism, decreased in tumor cells when the tumor cells were co-cultured with emetine-pretreated CAF (Figure 3D). Moreover, whereas the level of glutathione in tumor cells increased when they were co-cultured with non-treated CAF compared to in monoculture, it did not increase when they were co-cultured with emetine pretreated CAF (Figure 3E). Addition of 2 μM glutathione to the culture media prevented emetine-induced death of the tumor cells (Figure 3F). These combined data indicated that emetine inhibited the promotion of glycolysis and the provision of glutathione to tumor cells that are mediated by CAF, which resulted in a decreased potential of CAF to support tumor cells.

### The mechanism of action of emetine on tumor cells

**Figure 4:**
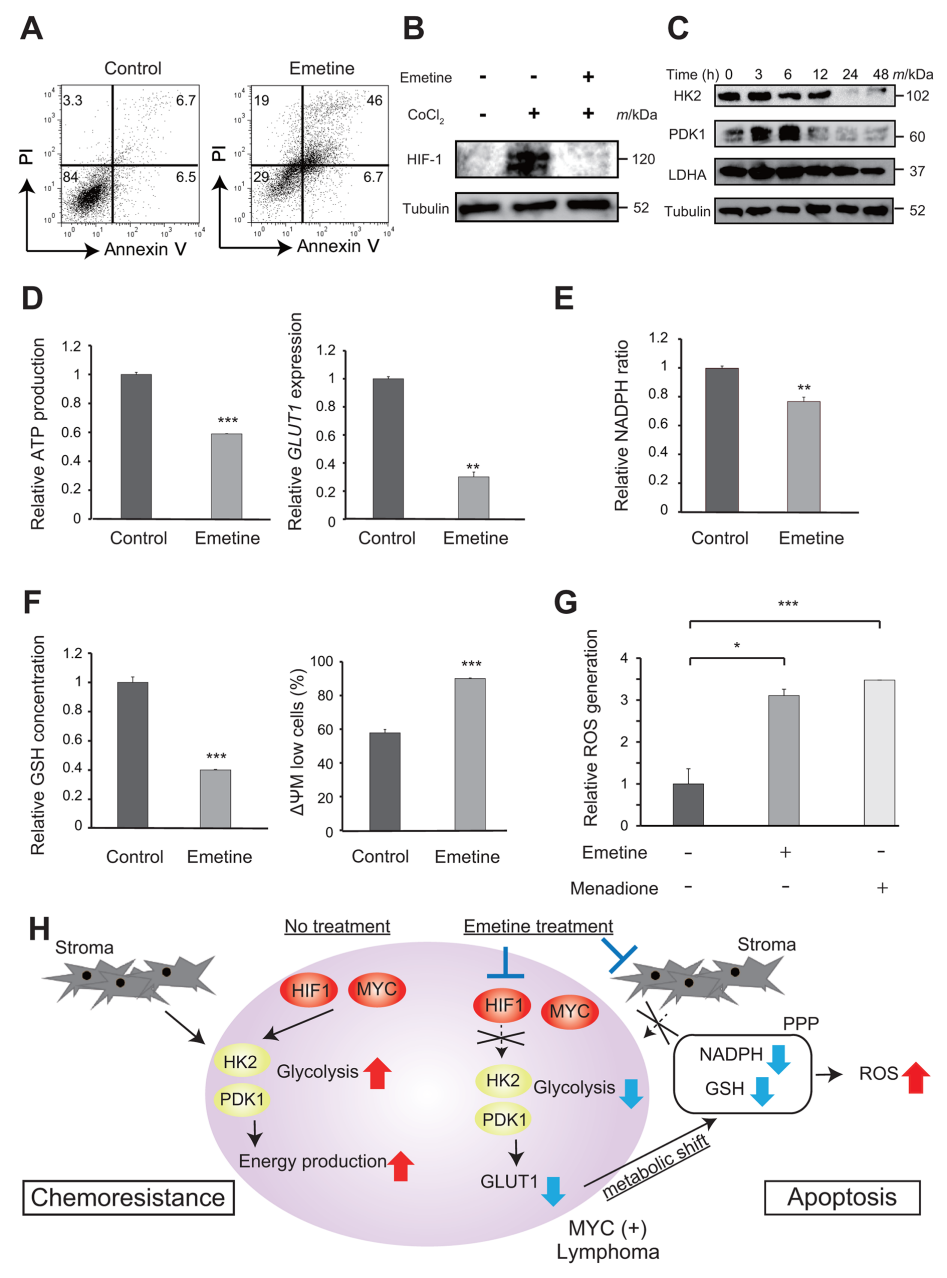
The mechanism of action of emetine on tumor cells **A**. Cell death of tumor cells (#2) co-cultured with CAF treated with emetine. Representative results of FACS analysis of the death of control cells and of cells treated with 0.5 μM emetine for 48 h are shown. **B**. Immunoblotting analysis of HIF-1α. Whole cell lysates of lymphoma cells (#1) were obtained 24 h after treatment with 100 μM CoCl_2_ with or without 0.5 μM emetine and were immunoblotted for HIF-1α. Tubulin was immunoblotted as a loading control. **C**. Immunoblotting analysis of key glycolytic enzymes in lymphoma cells (#2) in the presence of emetine. Whole cell lysates were obtained 0, 3, 6, 12, 24, and 48 h after treatment with 0.5 μM emetine and were immunoblotted for HK2, PDK1 and LDHA. Tubulin was immunoblotted as a loading control. **D, E, F, G**. Alteration of glucose metabolism of lymphoma cells (#2) in the presence of emetine. **(D) (left)** ATP production in lymphoma cells treated with emetine. Cell lysates were obtained 24 h after treatment of the co-culture of lymphoma cells with CAF with 0.5 μM emetine, and ATP production was then assessed relative to control. (right) Gene expression of the glucose transporter, *GLUT1* in lymphoma cells treated with emetine. RNA was extracted 12 h after treatment of the co-culture of lymphoma cells with CAF with 0.5 μM emetine, following which *GLUT1* mRNA expression relative to control was assessed using quantitative RT-PCR. **E**. The intracellular NADPH/NADP ratio in lymphoma cells treated with 0.5 μM emetine for 24 h is shown. **(F) (left)** The intracellular GSH concentration of lymphoma cells treated with 0.5 μM emetine for 24 h is shown. (right) The mitochondrial membrane potential (ΔΨM) of lymphoma cells treated with 0.5 μM emetine for 12 h is shown. Lymphoma cells were labeled with JC-1 reagents and were analyzed using a flow cytometer. The percentage of low ΔΨM cells was plotted on a bar graph. **(G)** ROS production of non-treated lymphoma cells (charcoal gray bars) or lymphoma cells treated with 0.5 μM emetine for 24 h (gray bars) or with 50 μM Menadione, which was used as a ROS inducer, for 3 h (pale gray bars) is shown. ROS production was measured using CellROX Green Oxidative Stress Reagents and is plotted on a bar chart. For D-G, each point represents the mean value taken from two (**D**) or three (**E, F, G**) independent experiments. Error bars indicate SEM. Asterisks indicate the *P* value as follows; *P < 0.05, **P < 0.01, ***P < 0.001. **H**. Scheme of the proposed mechanism of action of emetine. Emetine is proposed to induce apoptosis via alteration of glucose metabolism including glycolysis and the pentose phosphate pathway (PPP).

We next investigated mechanisms of emetine induced cell death in the tumor cells. Cell cycle analysis after emetine treatment revealed a population of sub G1, apoptotic cells in lymphoma cells co-cultured with CAF, which resulted from induction of a G2/M arrest (Figure 4A and Supplemental Figure S3A and S3B). Since it has been reported that emetine suppresses the expression of HIF-1α, which is a key regulator of glucose metabolism, [26–29] we evaluated HIF-1α expression in the tumor cells using western blotting. [30, 31] HIF-1α expression in the tumor cells was suppressed by treatment of the cells with 0.5 μM emetine in the presence of CoCl_2_, which was used to mimic a hypoxia condition and by treatment of cells grown under the condition of 5% O_2_ hypoxia for 48 h (Figure 4B and Supplemental Figure S3C). Taking into account the fact that emetine inhibited the promotion of glycolysis in tumor cells that was mediated by CAF as described above, we considered that emetine might suppress glycolysis in tumor cells, which would result in apoptosis of the tumor cells. As expected, the expression of enzymes involved in glycolysis was suppressed in the tumor cells in the presence of emetine under a hypoxia condition (Figure 4C and Supplemental Figure S3C). ATP production and both the mRNA and protein expression of GLUT1 in tumor cells were also suppressed by emetine treatment (Figure 4D and Supplemental Figure S3C). The serial signaling cascade that occurs following alteration of glycolysis, including decreased mitochondrial membrane potential, alteration of the pentose phosphate pathway and reduction in NADPH and glutathione that leads to the accrual of reactive oxygen species (ROS) and apoptosis accompanied by caspase cleavage, was observed in the presence of emetine (Figure 4E~4G and Supplemental Figure S3D). [32] These data indicated that emetine inhibited glycolysis in the tumor cells leading to the accumulation of intracellular ROS, which resulted in induction of apoptosis of the tumor cells (Figure 4H).

### *In vivo* efficacy of emetine

**Figure 5:**
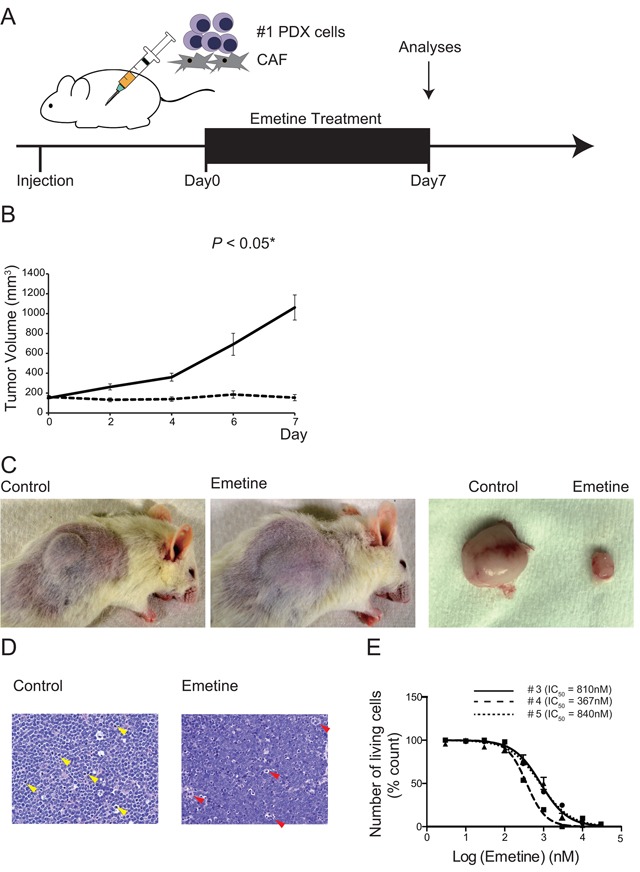
*In vivo* effect of emetine on intractable lymphoma cells **A**. The scheme of the experiment is shown. A mixture of 1 × 10^7^ lymphoma cells (#1) and 5 × 10^6^ CAF cells was subcutaneously inoculated into NOD/SCID mice. Mice were treated daily with emetine (N = 7) or vehicle (N = 7) intraperitoneally for 7 days. Tumor volume was measured and the mice were killed and analyzed on day 7. **B**. Tumor volumes of emetine (dotted-line) and control (solid line) treated mice were plotted on a line chart. Error bars indicate SEM. Asterisks indicate the *P* value as follows; *P < 0.05. **C**. Representative photographs of a vehicle treated mouse (left), an emetine-treated mouse (center), and resected tumors (right) on day 7 are shown. **D**. Representative HE staining of pathological specimens of mice killed on day 7 after initiation of treatment is shown. Mitotic cells observed in the control specimen are highlighted by yellow arrowheads (left). Degenerative cells observed in the emetine treated specimen are highlighted by red arrowheads (right). Original magnification × 400, using a Keyence BZ9000. **E**. Assessment of the death of lymphoma cells derived from DLBCL with *MYC* rearrangement. The viability of lymphoma cells from patient #3 (circles, solid line), #4 (squares, dashed line), and #5 (triangles, dotted-line) that were co-cultured with CAF in the presence of various concentrations of emetine for 48 h is shown. Each point represents the mean value for three independent experiments. Error bars represent SEM.

Finally, we evaluated the growth inhibitory effect of emetine on lymphoma cells *in vivo*. We subcutaneously injected 1 × 10^7^ PDX tumor cells originating from Pt #1 together with 2 × 10^5^ CAF into NOD/SCID mice. Inoculation of tumor cells with CAF ensured tumor cell engraftment according to our preliminary experiments. Once the mice had developed a subcutaneous tumor of at least 100 mm^3^ in size, we intraperitoneally administered 10 mg/kg of emetine as a treatment arm (N = 7) and dimethyl sulfoxide as a control arm (N = 7) for 7 days (Figure 5A). Emetine significantly inhibited the growth of the tumors compared to the control arm (p < 0.05) (Figure 5B and 5C). Body weight loss did not occur in either the treatment or the control arms (Supplemental Figure S4A). Representative pathological specimens stained with hematoxylin and eosin (HE) are shown in Figure 5D. Tumor cells undergoing mitosis were prominent in the control specimen, while degenerative cells were conspicuous in the tumor tissue treated with emetine. Consistent with this finding, 80% of the tumor cells in the control specimen were Ki67 positive in immunohistochemical (IHC) staining, while only 25% of the tumor cells in the treated specimens were Ki67 positive (Supplemental Figure S4B). Fibroblasts that stained with α-SMA in IHC were observed in the control specimen; however, we also observed a small number of α-SMA positive fibroblasts in the treated specimen, indicating that emetine also suppressed the growth of CAF *in vivo* (Supplemental Figure S4B). Moreover, we investigated the effect of emetine on the primary tumors of three other patients who were diagnosed with DLBCL with *MYC/BCL2* rearrangement (Supplemental Table S2). We confirmed that the efficacy of emetine for these cells was within the same range as that for the samples from Pt # 1 and #2, with an IC_50_ ranging from 367 to 840 nM (Figure 5E). These data indicated that emetine was potentially useful for the treatment of intractable *MYC* related lymphomas.

## DISCUSSION

We successfully identified the drug, emetine, which was effective against intractable DLBCL with *MYC* rearrangement, through the use of a high-throughput drug screening system using primary patient tumor cells. In the subsequent analyses of the mechanism of action of emetine, we found that the anti-tumor effect of this drug is mediated by its effect on the interaction between tumor cells and stromal cells in the tumor microenvironment. Moreover, we successfully developed CAF from human lymph nodes, which were useful for drug screening. Our system, which is based on human-origin cells, identified a drug that was effective in targeting a tumor microenvironment, which could lead to the development of a novel treatment for intractable lymphoma.

Emetine has been approved as an anti-protozoal drug and as an emetic, and has been used in a clinical setting all over the world. We determined the dose of emetine as 10 mg/kg in mice in the *in vivo* analyses, which is equivalent to a dose of 0.8 mg/kg in humans. This dose is lower than the approved dose of 1 to 10 mg/kg in a clinical setting in humans [33]. Emetine-induced toxicities, including cardiac toxicities, have sometimes been reported in the clinical use of emetine in humans. However, the current dose of 0.8 mg/kg determined in the present study is safe in humans, with a low incidence of severe toxicities. The 50% of lethal dose in the mouse has been established as 16.2 mg/kg, and we confirmed that the same or higher dose was quite harmful for mice. We thus considered that it was inappropriate to administer emetine to mice at a higher dose than 10 mg/kg with the expectation of further anti-lymphoma effects. Although it should be clarified whether a dose of 0.8 mg/kg emetine demonstrates anti-lymphoma effects in humans, we believe that emetine could elicit a treatment effect at a dose at which it exerts minimum toxicity. Further investigation of the efficacy of emetine is warranted.

MYC and HIF-1α are known to cooperatively activate glycolysis to generate adequate energy for tumor cells, resulting in chemo-resistance through the upregulation of many genes relevant to glucose metabolism [27, 34, 35]. In the present analysis, emetine affected both CAF and the tumor cells *in vitro*; it inhibited CAF-mediated support of tumor cell survival and it inhibited glycolysis due to the suppression of HIF-1α and key enzymes of glycolysis including HK2 and PDK1 in tumor cells, leading to the accumulation of intracellular ROS and the induction of apoptosis. Considering that emetine tended to be more effective in lymphoma with *MYC* rearrangement than in lymphoma without *MYC* rearrangement, emetine could be highly effective in tumor cells with increased glycolysis. In addition, it should be mentioned that HIF-1α is a potential therapeutic target. Thus, considering the molecular basis of HIF-1α activity, an HIF-1α inhibitor is also an attractive drug. Indeed, we identified an HIF-1α inhibitor, chetomin, as a promising anti-lymphoma drug candidate with an IC_50_ of 1.3 nM (Supplemental Table S1). However chetomin could not be applied to humans due to severe toxicity [36, 37]. The development of tumor specific HIF-1α inhibitor, which has a reduced off-target effect compared to chemotin, is warranted.

In the present study, using a drug screening method we found that emetine might be a promising drug against *MYC*-associated intractable DLBCL. We believe that targeting the tumor microenvironment is a potentially promising strategy for the treatment of current refractory diseases. However, careful interpretation of potential clonal selection and/or evolution of tumor cells during development of the CAF and PDX models are required. The CAF that we used in this study were not derived from the same patient whose tumor cells we evaluated. Mutual interaction between CAF and tumor cells might differ compared to when both CAF and tumor cells are derived from the same patient. Moreover we only evaluated the interaction of tumor cells with CAF in this study. Needless to say, the tumor microenvironment also includes other cells such as T cells, B cells, macrophages, and fibroblast dendritic cells. Thus, we could only study some of the interactions of tumor cells with the tumor microenvironment. Nevertheless, a search for effective drugs through a screening method that uses primary tumor samples and/or tumor cells from the PDX model might be a quite promising avenue for the development of a novel treatment strategy against intractable disease. Validation of the effectiveness of the drug identified in this study is warranted in order to confirm the utility of our method.

## MATERIALS AND METHODS

### Patient samples

All patient samples were collected from patients diagnosed with lymphoma and were used experimentally after obtaining written informed consent. The study protocol for the experimental use of patient samples and information was approved by the institutional review board of Nagoya University Hospital and complied with all provisions of the Declaration of Helsinki and the Ethical Guidelines issued by the Ministry of Health, Labour and Welfare in Japan.

### Establishment of PDX cells and CAF

PDX cells were established as described previously [24, 38]. In brief, to develop xenograft mouse models, 1.0 × 10^6^ ~ 5.0 × 10^6^ of tumor cells from patients pathologically diagnosed with DLBCL were transplanted intravenously into NOD/Shi-*scid* IL2Rγ^null^ (NOG) mouse (purchased from the Central Institute for Experimental Animals, Tokyo, Japan). To suppress the proliferation of human T cells from the patient tumor in NOG mice, 100 μg of OKT3, an anti-CD3 monoclonal antibody (mAb), were also injected intraperitoneally (BioLegend, San Diego, CA, USA). The engraftment of lymphoma cells was investigated using flow cytometry and pathological specimens. All the animal experimental procedures complied with the Regulations on Animal Experiments in Nagoya University.

CAFs were established as follows. A fresh patient lymph node sample was mashed to obtain a cell suspension for subsequent diagnostic analyses. The residue was then loosened in 0.25% trypsin-ethylendiaminetetraacetic acid solution and cultured in Iscove's Modified Dulbecco's Medium (IMDM) (Sigma-Aldrich, St. Louis, MO, USA) supplemented with 10% fetal bovine serum (FBS) (Gibco in Thermo Fisher Scientific, Waltham, MA, USA). Of the various types of cells in this culture, only the spindle-shaped adherent cells survived for more than several months. These spindle-shaped adherent cells show the expression of α-SMA positive and CD31 negative. Since such adherent cells were not established from benign disease samples, these patient-derived adherent cells were regarded as CAF. These CAF were maintained in the above-mentioned culture condition by subculture once a week.

### Drugs, compound library and cell lines

Emetine dihydrochloride hydrate and menadione were purchased from Sigma-Aldrich. Reduced-form glutathione (GSH), 4-hydroperoxy cyclophosphamide and CoCl_2_ were purchased from Wako Pure Chemical Industries (Osaka, Japan), Toronto Research Chemicals (Toronto, Canada) and Kanto Chemical (Tokyo, Japan), respectively. Library compounds mainly consisting of off-patent drugs and pharmacologically active reagents were provided by the Drug Discovery Initiative (The University of Tokyo, Tokyo, Japan) as described previously [24]. SU-DHL4, SU-DHL10, OCI-Ly3 and OCI-Ly10 cell lines were kindly provided by Dr. Kunihiko Takeyama (Dana Farber Cancer Institute, Boston, MA, USA) within the context of collaboration. BLS4 cells were kindly provided by Dr. Tomoya Katakai (Niigata University, Niigata, Japan). SU-DHL6, Raji and Daudi cell lines were obtained from the ATCC (Manassas, VA, USA). The RRBL-1 cell line was established in our laboratory [39].

### Cell proliferation assays

To evaluate cell proliferation, the cells were seeded in six-well plates (5 × 10^4^ cells per well) and were cultured at 37 °C in a 5% CO_2_ incubator. Cell numbers were counted every 24-48 h over a 7 day period. Cell counts were accurately measured with the TC20 automated cell counter (Bio-Rad, Hercules, CA, USA). For the WST-8 assay, the cells were seeded in 96-well plates and were cultured for 72 h. Ten microliters of the Cell Counting Kit-8 reagent (Dojindo Laboratory, Kumamoto, Japan) was then added into each well and fluorescence was evaluated at 450 nM using the GloMax^®^-Multi Detection System (Promega, Madison, WI, USA).

### Cell death and cell cycle assessment

To evaluate the cell death of tumor cells co-cultured with CAF, we assessed cell death using an image analyzer as reported previously [24]. In brief, 1 × 10^3^ CAF were placed in 96-well plates and were incubated for 24 h. Subsequently, 3 × 10^4^ tumor cells were added into each well and the cells were co-cultured for 24 h. The appropriate drug was then added into each well, and, after 48 h incubation, total and dead cells were stained with Hoechst 33342 and 15 μg/ml of propidium iodide (PI). Dead lymphoma cells were selectively counted with an Array Scan VTI HCS Reader (Thermo Fisher Scientific). To evaluate cell death of mono-cultured tumor cells, a PI and Annexin V-fluorescein isothiocyanate (FITC) assay was performed as described in detail previously [40, 41]. In brief, cells were seeded in 96-well plates, were incubated with the required drugs for 48 h and were then stained with 10 μg/mL PI and 10 μg/mL Annexin V-FITC for 15 min at room temperature in the dark. Cell death was assessed using flow cytometry (FACSCalibur, BD, Franklin Lakes, NJ, USA) and was analyzed using FlowJo Version 7.6.5 software (TreeStar, Ashland, OR, USA). For cell cycle assessment, cells were assessed using the hypotonic PI assay that was described in detail previously [40]. Cells were incubated with the appropriate drugs for 12 h, were then washed and re-suspended in phosphate-buffered saline (PBS) containing 0.2% Triton X-100 and 50 μg/mL PI before analysis using flow cytometry. Data were analyzed with ModFit LT cell-cycle analysis software (Verity Software House, Topsham, ME, USA).

### Drug screening

To extract effective anti-tumor drugs from library compounds through high-throughput drug screening, we assessed cell death based on co-culture methods using CAF as mentioned above. For this screening, 2 μM of one of the 3,440 library compounds was added into each well and was incubated for 48 h. All screenings were performed with Z’-factors > 0.5 and coefficient of variation values < 10%, demonstrating the suitability for screening. The effect of each library compound on tumor proliferation on CAF was measured using the WST-8 assay as described above.

### Measurement of tumor metabolic products

ROS production was assessed using a fluorogenic probe (CellRox Deep Red Reagent, Thermo Fisher Scientific) as described previously [24]. Adenosine triphosphate (ATP) concentration was assessed using the Colorimetric ATP Assay Kit (Abcam, Cambridge, UK). ATP generation was normalized by the number of cells. The GSH concentration was calculated using the GSH-Glo Assay kit (Promega). Cellular NADPH contents were determined by a colorimetric determination method using an NADP/NADPH determination kit (Biovision, Milpitas, CA, USA). Mitochondrial membrane potential was measured by using the JC-1 Mitochondrial Membrane Potential Assay Kit (Cayman Chemical, Ann Arbor, MI, USA). All procedures were conducted according to the manufacturer's instructions.

### Immunoblotting

Immunoblotting was performed as described previously [40]. In brief, cells were treated with the indicated drug and lysed. Samples were separated by sodium dodecyl sulfate polyacrylamide gel electrophoresis and transferred to polyvinyldene difluoride membranes that were then blocked with 5% skimmed milk in TBS-Tween buffer (50 mM Tris-HCL [pH 7.4], 150 mM NaCl and 0.05% Tween 20). Immunoblotting was carried out using primary antibodies (Supplementary Table S3) and signals were detected with the appropriate horseradish peroxidase–conjugated second antibodies. Images were visualized with the LAS-4000 mini image analyzer (Fujifilm, Tokyo, Japan) and analyzed with MultiGauge software (Fujifilm).

### Quantitative real-time reverse transcriptase (RT)-PCR analysis

RNA was extracted from cell lysates (RNeasy Mini Kit, Qiagen, Venlo, Netherlands) and complementary DNA was prepared with SuperScript II Reverse Transcriptase and random primers (Thermo Fisher Scientific). Quantitative PCR analysis was conducted as previously described [42]. Quantitative RT-PCR analysis of the expression of *GLUT-1* was performed with 40 cycles of two-step PCR (15 seconds at 95 °C and 60 seconds at 60 °C) after initial denaturation (50 °C for 2 min and 95 °C for 10 min) using an Applied Biosystems 7300 Real-Time PCR System (Applied Biosystems in Thermo Fisher Scientific). Data were normalized by the amount of *HPRT1* mRNA using gene-specific primers (Supplementary Table S4).

### Pathological analyses, immunohistochemical staining and fluorescence in-situ hybridization

The FFPE tissues of patient and mouse samples were evaluated using routine HE and IHC staining. Chromosomal G-banding was performed by the LSI Medience Corporation (Tokyo, Japan). Break-apart of the chromosome at *MYC* and *BCL2* genes was also routinely evaluated by FISH analysis that was performed in our pathology department. L26 (Dako, Glostrup, Denmark) was used as the mAb targeting CD20. IHC for CD20 was performed as described previously [38]. For IHC of α-SMA (Dako) and Ki-67 (Dako), after deparaffinization and rehydration of the sections, antigen retrieval was performed in Target Retrieval Solution, Citrate pH 6 (Dako) for 15 minutes at 98 °C using a microwave oven and for 5 minutes at 121 °C using an autoclave, respectively. The sections were subsequently incubated with primary antibody at 4 °C overnight followed by the addition of biotin-conjugated secondary antibody for 2 h at room temperature. Staining was activated by addition of the avidin-biotin complex (ABC). Horseradish peroxidase activity was detected with 3, 3-diaminobenzidine tetrahydrochloride (DAB). The specimens were observed with an Olympus BX51 N-34 (Olympus, Tokyo, Japan), and the photographs were taken with a BZ9000 (Keyence, Osaka, Japan). All pathological specimens were reviewed by hematopathologists (S.S. and S.K.) according to the current WHO classification.

### *In vivo* studies

To evaluate the anti-lymphoma effect of emetine *in vivo*, 1 × 10^7^ tumor cells from patient #1 together with 2 × 10^5^ CAF were subcutaneously inoculated into the flank of NOD/SCID mice (purchased from CLEA Japan Inc. Tokyo, Japan). To suppress the proliferation of human NK cells from the patient's tumor in NOD/SCID mice, 500 μg of anti-asialo ganglio-N-tetraosylceramide (GM1) rabbit polyclonal antibody were also injected intraperitoneally (Wako Pure Chemical Industries). Treatment was initiated when the inoculated tumors reached a size of at least 100 mm^3^, defined as day 0. Seven mice were randomly divided into control and emetine treatment groups according to tumor volume. Mice were intraperitoneally treated daily with vehicle or 10 mg/kg/day of emetine for 7 days. Tumor volume was measured every day and was calculated using the following formula: Tumor volume (mm^3^) = (d^2^ × D)/2, where D (mm) and d (mm) are the longest and shortest diameters of the tumor, respectively.

### Statistical analysis

All quantitative results are presented as the mean ± standard error of the mean taken from two or three independent experiments. The statistical significance of *in vitro* experiments was evaluated by an unpaired t-test or by two-way ANOVA, and *P* < 0.05 was considered significant. Regarding *in vivo* analyses, the results were analyzed with repeated measure ANOVA with Tukey's multiple comparisons test to determine statistical significance at a significance level of *P* < 0.05. All statistical analyses were performed using GraphPad Prism Version 6 (GraphPad Software Inc., La Jolla, CA).

## SUPPLEMENTARY FIGURES AND TABLES




