# Supplementary material for: Emetine elicits apoptosis of intractable B-cell lymphoma cells with MYC rearrangement through inhibition of glycolytic metabolism
Source: Oncotarget. 2016 Dec 31;8(8):13085–98. doi: 10.18632/oncotarget.14393 (PMC5355079; doi:10.18632/oncotarget.14393)
Supplement: Supplementary file 1 [file oncotarget-08-13085-s001.pdf]

Emetine elicits apoptosis of intractable B-cell lymphoma cells with *MYC* rearrangement through inhibition of glycolytic metabolism

SUPPLEMENTARY FIGURES AND TABLES

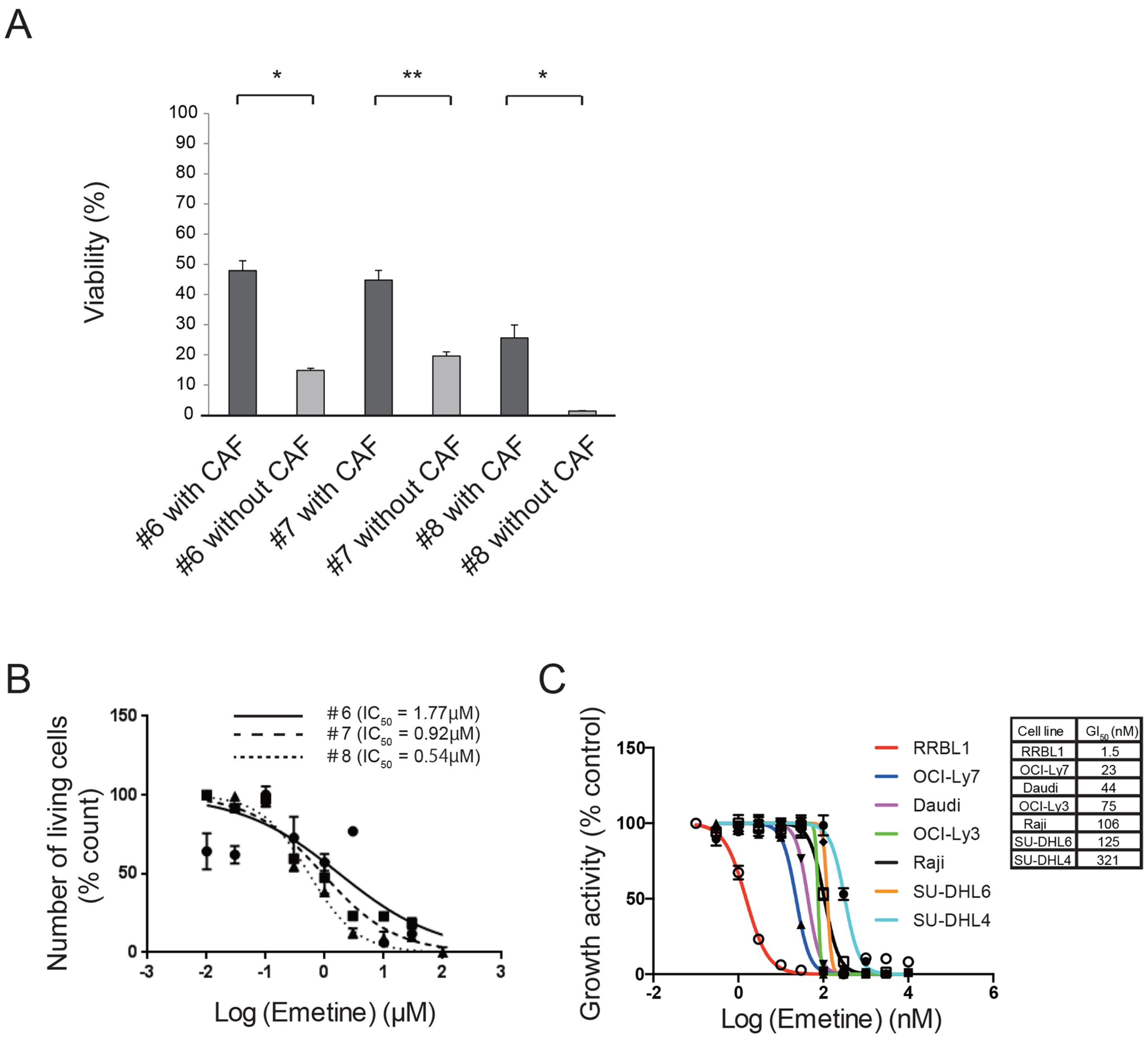

Supplementary Figure 1: The effects of emetine on various lymphoma cells. A. Viability of lymphoma cells without *MYC* rearrangement co-cultured with or without CAF. A bar graph of relative cell viability under each culture condition is shown. Each point represents the mean value taken from three representative independent experiments. Error bars indicate SEM. Asterisks indicate the P value as follows; * $P < 0.05$; ** $P < 0.01$. **B.** Assessment of the emetine-induced death of lymphoma cells derived from DLBCL without *MYC* rearrangement. The viability of lymphoma cells from patient #6 (circles, solid line), #7 (squares, dashed line), and #8 (triangles, dotted-line) that were co-cultured with CAF in the presence of various concentrations of emetine for 48 h is shown. Each point represents the mean value of three independent experiments. Error bars represent SEM. **C.** Inhibition of the growth of various DLBCL cell lines in the presence of various concentrations of emetine for 72 h is shown. Each point represents the mean value taken from three independent experiments. Error bars indicate SEM.

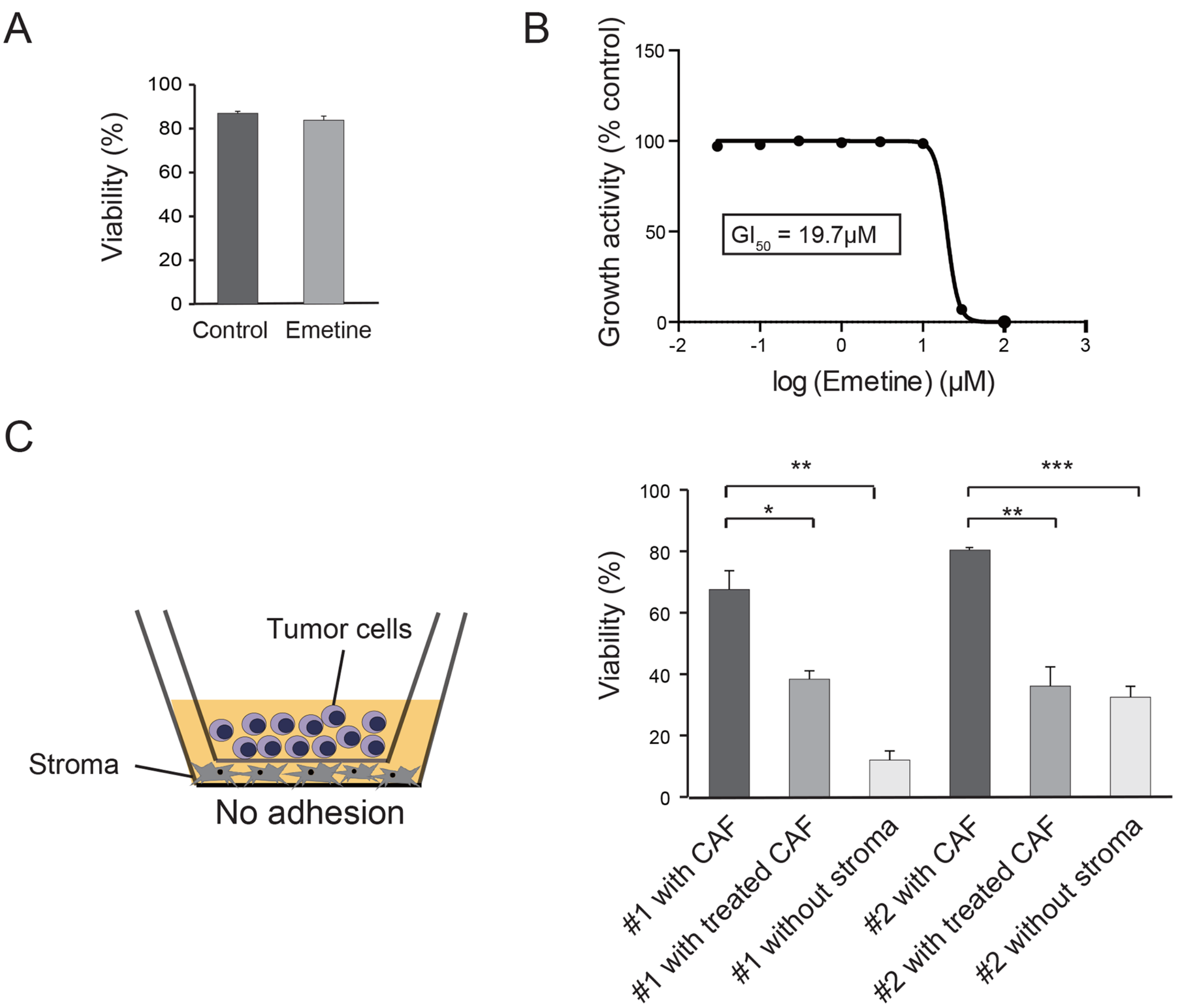

Supplementary Figure 2: The effects of emetine on cancer associated fibroblast (CAF). A. Assessment of the effect of emetine treatment on the death of CAF. Cell death was assessed 48 h after treatment with 2 μM emetine by counting dead cells stained with DAPI with an image analyzer. Each point represents the mean value taken from two independent experiments. Error bars indicate SEM. **B.** Inhibition of the growth of CAF in the presence of various concentrations of emetine for 72 h is shown. Each point represents the mean value taken from three independent experiments. Error bars indicate SEM. **C.** Assessment of the death of lymphoma cells co-cultured with CAF that were separated from the lymphoma cells by a transwell membrane. The death of lymphoma cells (#1 and #2) (3×10^4 per well) was assessed 48 h after co-culture with the separated CAF (3×10^3 per well) that were treated with or without 0.5 μM emetine for 48 h prior to the initiation of co-culture. Cell viability was assessed using the PI and Annexin V-FITC method. Each point represents the mean value taken from three independent experiments. Error bars indicate SEM. Asterisks indicate the P value as follows; *$P < 0.05$ ** $P < 0.01$, *** $P < 0.001$.

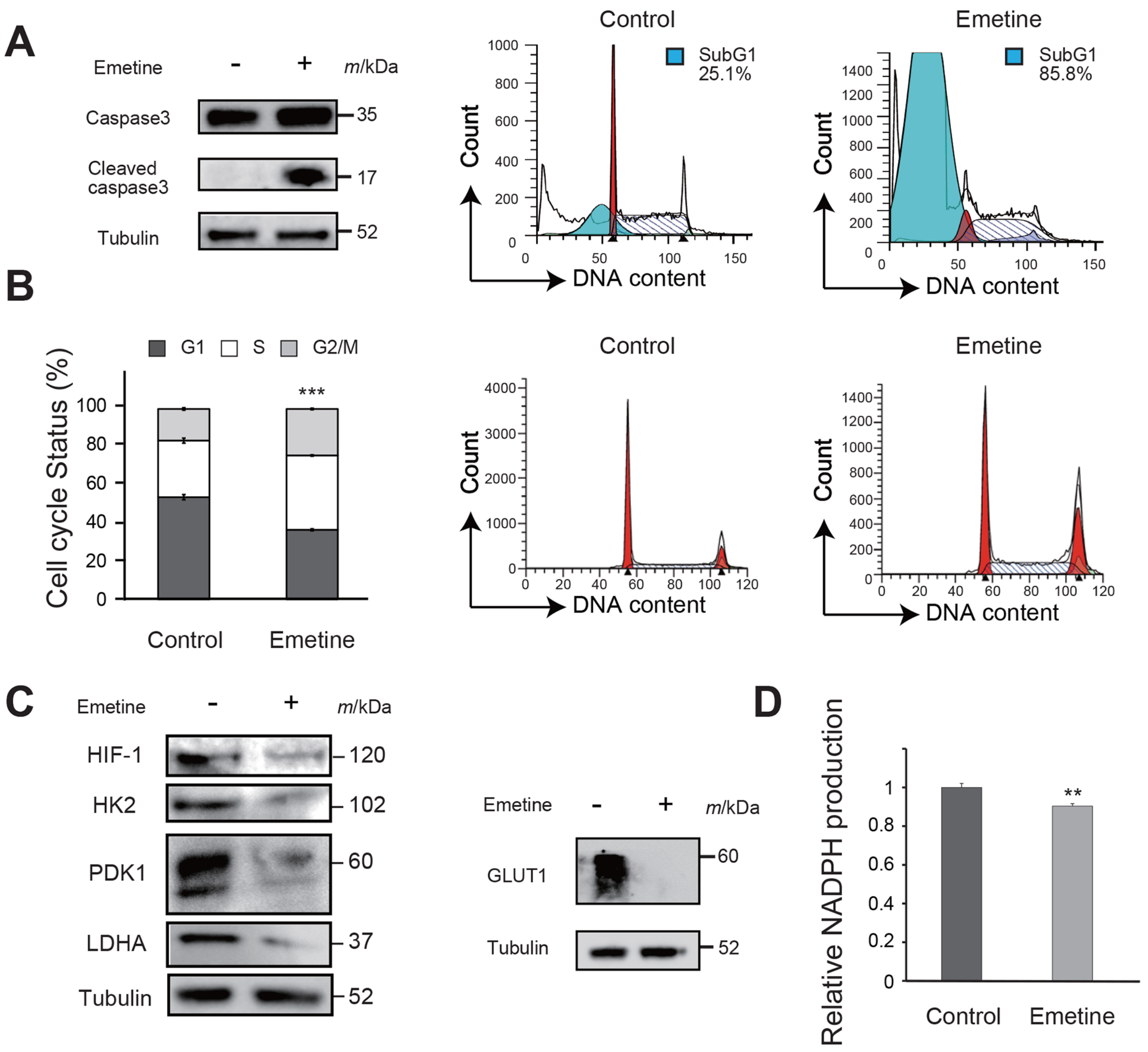

Supplementary Figure 3: Emetine induces apoptosis of lymphoma cells. A. Immunoblotting and cell cycle analyses of lymphoma cells (#2) co-cultured with CAF that were treated with emetine. Whole cell lysates were obtained 48 h after treatment with 0.5 µM emetine and were immunoblotted for Caspase 3 and Cleaved caspase 3 (left panel). Tubulin was immunoblotted as a loading control. Whole cell lysates obtained 48 h after treatment without (control) or with 0.5 µM emetine were lysed in hypotonic PI solution and analyzed using flow cytometry. The percentage of cells in the sub G1 cell cycle phase is indicated (middle and right panels). **B.** Cell cycle analyses of lymphoma cells (#2) co-cultured with CAF after emetine treatment. Lymphoma cells (#2) were treated with 0.5 µM emetine for 12 hours, and were then lysed in hypotonic PI solution and analyzed using flow cytometry. The percentage of cells in G0/G1, S and G2/M cell cycle phases are indicated (left panel). Bars represent the mean value of three independent experiments. Error bars represent SEM. Asterisks indicate the P value as follows; *** $P < 0.001$. Representative DNA content flow profiles of control and emetine-treated lymphoma cells are shown in the left and right panels, respectively. **C.** Immunoblotting analysis of lymphoma cells (#1) co-cultured with CAF under hypoxia condition (5% O2 hypoxia for 48 h) that were treated with emetine. Whole cell lysates were obtained 24 h after treatment with 0.5 µM emetine and were immunoblotted for HIF-1α, HK2, PDK1, LDHA (left panel) and GLUT1 (right panel). Tubulin was immunoblotted as a loading control. **D.** Intracellular NADPH levels in lymphoma cells co-cultured with CAF that were treated with 0.5 µM emetine for 24 h is shown.

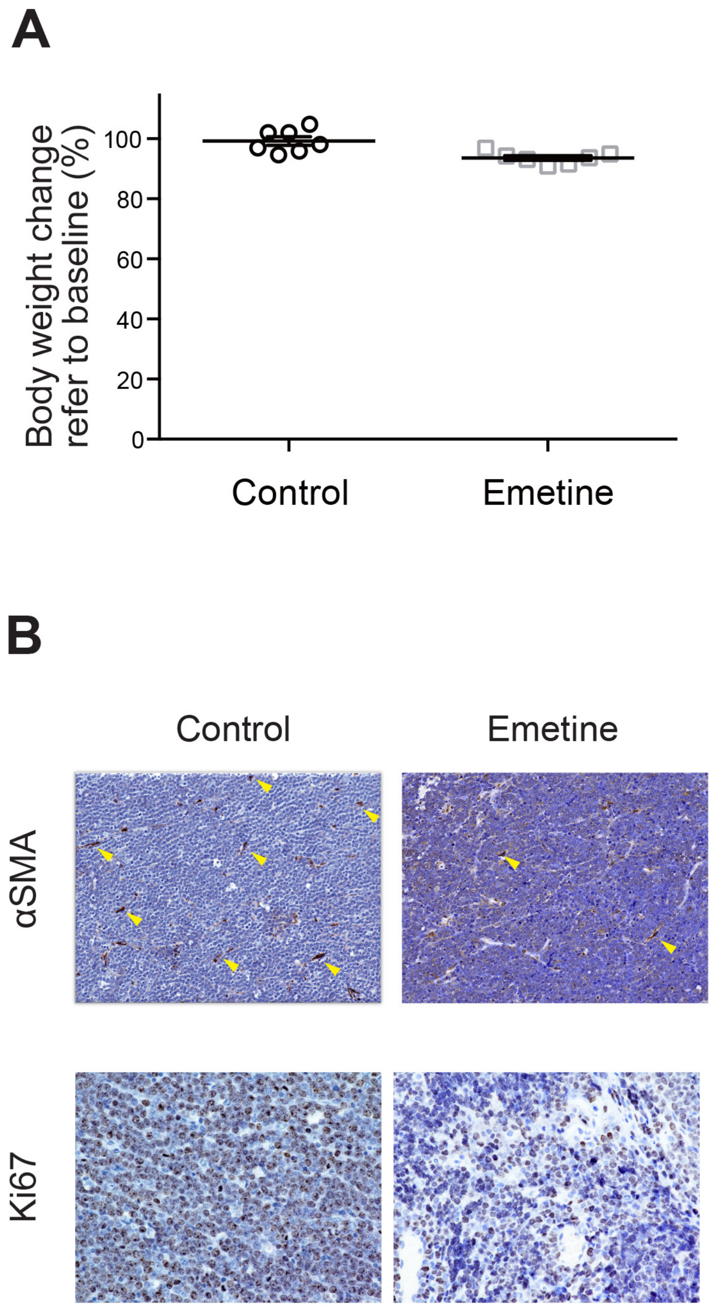

Supplementary Figure 4: *In vivo* effect of emetine. A. The change in body weight of the mice during the *in vivo* experiment is shown. Horizontal lines represent the mean value taken from seven mice. Error bars indicate SEM. **B.** Representative α-SMA and Ki67 immunostaining of pathological specimens of mice killed on day 7 after initiation of emetine treatment is shown. α-SMA positive fibroblasts (highlighted by yellow arrowheads) and Ki67 positive proliferating lymphoma cells are conspicuous in the control specimen (upper and lower left panels, respectively) but not in the emetine treated specimen (upper and lower right panels, respectively). Original magnification × 200 (upper panels),× 400 (lower panels), using a Keyence BZ9000.

Supplemental Table 1: Top 10 drugs selected by high-throughput 3440 drug screening

	Patient #1 viability (%)	Patient #2 viability (%)	Relative MTT value of stroma cells
Chicago sky blue 6B	0	0	1.13
Doxorubicin hydrochloride	0	0	1.3
Daunorubicin hydrochloride	0	0	0.92
Pyrvinium pamoate	0	0	1.09
Verteporfin	0	0	0.91
Chetomin	9.87	4.3	0.46
Celastrol	12.97	19.63	0.84
(+)-Brefeldin A	14.06	1.46	0.7
Proteasome Inhibitor I (Aldehyde)	15.46	28.61	0.47
Emetine	16.87	18.59	0.84

Supplemental Table 2: Characteristics of DLBCL patients #3, #4, #5, #6, #7 and #8

See supplementary File 1

Supplemental Table 3: Antibodies and fluorescent reagents used in this study

Antibody	Fluorochrome	Clone	Isotype	Company	Location
HIF-1α			Rabbit IgG	Cell Signaling Technology	Danvers, MA, USA
HK2		C664G5	Rabbit IgG	Cell Signaling Technology	Danvers, MA, USA
PDK1		D37A7	Rabbit IgG	Cell Signaling Technology	Danvers, MA, USA
LDHA		C4B5	Rabbit IgG	Cell Signaling Technology	Danvers, MA, USA
Glut1		D3J3A	Rabbit IgG	Cell Signaling Technology	Danvers, MA, USA
Caspase-3		8G10	Rabbit IgG	Cell Signaling Technology	Danvers, MA, USA
α-tublin		Ab-1	Mouse IgG	Millipore	Billerica, MA, USA
Annexin V	FITC			Medical & Biological Laboratories	Aichi, Japan
PI				Sigma-Aldrich	St. Louis, MO, USA
Hoechst33342				Sigma-Aldrich	St. Louis, MO, USA

Supplemental Table 4: Gene specific primer used for quantitatiive RT-PCR

Gene name	Assay ID	Taqman Part Number
solute carrier family 2 (facilitated glucose transporter), member 1	Hs00892681_m1	4331182
hypoxanthine phosphoribosyltransferase 1	Hs02800695_m1	4448489
